# Breathing Reserve and Lung Function in Female Elite Runners

**DOI:** 10.3390/sports13070231

**Published:** 2025-07-14

**Authors:** Ferdinand Grov Kyte, Karoline Holsen Kyte, Linn Skinstad, Jonny Hisdal, Trine Stensrud

**Affiliations:** 1 Institute of Clinical Medicine, Faculty of Medicine, University of Oslo, 0318 Oslo, Norway; f.g.kyte@studmed.uio.no (F.G.K.); karolineholsenkyte@gmail.com (K.H.K.); jonny.hisdal@medisin.uio.no (J.H.); 2 Department of Vascular Surgery, Oslo University Hospital, Aker, 0586 Oslo, Norway; 3 Department of Sports Medicine, Norwegian School of Sport Sciences, 0806 Oslo, Norway; linn.skinstad@hotmail.no; 4 Sedsvoll Skole, 2072 Dal, Norway

**Keywords:** breathing reserve, ventilatory reserve, female elite runners, female elite athletes, lung function, VO_2_max

## Abstract

Breathing reserve (BR) is the remaining proportion of achievable minute ventilation that remains unutilized at total exhaustion during exercise. Previous studies have found a smaller BR in endurance-trained athletes compared to untrained controls. However, most of these studies have examined men. Given that women have a greater ventilatory limitation than stature-matched men, the present cross-sectional study aimed to investigate how this sex difference influences BR and lung function tests in endurance-trained females compared to matched, untrained females. To obtain further insight, we also aimed to investigate whether VO_2_max serves as a predictor of BR. We examined 15 female elite runners and 15 healthy, matched female controls aged 24–33 years with regard to pulmonary function, MVV, V_E_max, BR, and VO_2_max. The elite runner group had a median BR of 5%, while that of the controls was 21%, representing a significant difference. Lung function tests showed no differences between the two groups. A moderate association was found between VO_2_max and BR. The female elite runners demonstrated a lower BR than the group of matched, untrained controls, which was lower than that found for male elite athletes in previous studies. This may indicate a greater ventilatory demand in female relative to male endurance athletes.

## 1. Introduction

The human body has an impressive extra ventilatory capacity, with healthy people usually being able to increase a normal ventilatory minute volume of approximately 6 L by at least 17–20 times [1]. Despite this achievable minute ventilation, most healthy people never fully utilize this potential, even during heavy exercise. This is because, for most people, the cardiovascular system is the major part limiting their maximal oxygen uptake (VO_2_max) [2]. Studies have shown that healthy people usually use only 60–80% of their ventilatory capacity during heavy exercise [3,4,5,6], leaving an unused breathing reserve (BR) of 20–40%. BR represents the proportion of an individual’s estimated or measured maximal ventilatory capacity that is not utilized during peak physical exhaustion. The ventilation measured at peak exercise is referred to as maximal minute ventilation (V_E_max), while the estimated or measured upper limit of minute ventilation is normally defined as maximal voluntary ventilation (MVV) [5,7,8]. MVV can be calculated or measured in different ways, which may influence the estimated BR [8]. Endurance training improves the cardiovascular system’s capacity for O_2_ transport, but a comparable adaptation is not seen in the pulmonary system [9,10]. Therefore, the cardiovascular system may not be the limiting factor in the O_2_ transport chain of highly endurance-trained people, and they may need to utilize a greater proportion of their lung capacity than untrained individuals. Previous studies support this hypothesis, finding that a BR of 5–15% during heavy exercise is normally observed in endurance-trained athletes [6,11,12,13]. There is undoubtedly a difference in BR between the two groups when comparing different studies, but very few studies have investigated the correlation between VO_2_max and BR. Therefore, it is unknown whether VO_2_max can serve as a predictor of BR, or, further, whether BR can at some point become so low that it might indicate a ventilatory limitation to VO_2_max.

Women have a greater ventilatory restriction than men, even when adjusted for stature [1]. Both lungs and airways are relatively smaller in women compared to men [1,14,15,16,17,18,19]. This demands harder work from the ventilatory muscles and can potentially require the utilization of a greater proportion of their ventilatory reserve. According to McArdle et al., it may result in a smaller BR for women than for men in general, and an even greater difference among well-trained women [1]. Hence, ventilation could be a more common limiting factor for VO_2_max in endurance-trained women than men. However, there is a lack of previous research on BR in study populations consisting exclusively of well-trained women, particularly elite runners, and the same applies to healthy, untrained women.

Furthermore, it is widely acknowledged that lung function, unlike both the cardiovascular and neuromuscular systems, shows no significant endurance training–induced increase in functional capacity [1,20,21]. However, some studies have demonstrated that this may not be entirely true, with forced expiratory volume in the first second (FEV_1_) and forced vital capacity (FVC) emerging as parameters that may improve with increased physical activity [22,23,24]. This divergence indicates that further research is needed to examine this matter.

The main aim of the present study is therefore to compare BR between a group of female elite runners and a matched group of healthy, untrained female controls. Based on previous studies, our hypothesis is that the runners will have a significantly lower BR than the controls. Secondarily, we hypothesize that VO_2_max could serve as a predictor of BR. Finally, our study aims to investigate whether there is a difference in lung function, especially FEV_1_ and FVC, between the two groups. The hypothesis is that there will be no difference between the groups, in line with the current consensus.

## 2. Materials and Methods

The present study is a cross-sectional controlled study, and all data were collected simultaneously with data collected for two other previously published studies on vascular function and bone health in the same study population [25,26]. The recruitment process took place during the period from October 2019 to January 2020 through announcements on social media and the distribution of written information about this study to relevant candidates. Those receiving the latter were female long-distance and trail runners among Norway’s top 20, based on statistics from 2019. Students at the University of Oslo were recruited as the control group, consisting of matched healthy, untrained women. For both groups, the inclusion criteria were aged 18–35 years, non-smoking, nulliparous, and otherwise healthy. In addition, a minimum of 8 h of endurance training per week was required for the group of runners, while the control group had a requirement of a maximum of 2 h of training weekly. Any regular medication, except oral contraceptives, was not permitted. Initially, 16 female runners and 17 inactive women were included. However, due to injuries and problems concerning the then-ongoing COVID-19 situation, 3 participants withdrew, and the final study sample included in the analysis consisted of 15 runners and 15 controls. They all signed written informed consent forms. All participants were Caucasian, and the age range was 24–33 years. Our study received approval from the Regional Committees for Medical and Health Research Ethics (REC Southeast, reference 2019/155).

Initially, height and weight were measured, and information about training volume was collected. To determine fat mass and fat-free mass, a DXA scan (Lunar, Prodigy Densitometry, GE Medical Systems, Chicago, IL, USA) was conducted. Thereafter, participants performed lung function measurements, including spirometry and MVV, and ultimately an exercise test was performed to determine maximal oxygen uptake (VO_2_max) and V_E_max. All participants were carefully instructed on all tests before performing them.

Pulmonary function tests were conducted using the MasterScreen PFT (Jaeger, CareFusion, Höchberg, Germany), according to the guidelines from the American Thoracic Society (ATS) and the European Respiratory Society (ERS) [7,27]. Participants were examined separately in a seated position while wearing a nose clip. They were instructed to take a few tidal breaths, inhale maximally, and then exhale as forcefully and quickly as possible until they felt no more air could be expelled. This was performed at least three times until two valid performances with less than 5% difference in FEV_1_ were observed. FEV_1_ and FVC were determined from the highest measured values among the valid attempts, and FEV_1_/FVC was subsequently calculated. All values were expressed both in absolute values and relative to the predictive reference values according to the Global Lung Initiative (GLI) 2012, based on height, ethnicity, age, and sex [28,29]. An online GLI-2012 calculator was used for this purpose [30], which also provided the Z-score and the lower limit of normal (LLN).

For measuring MVV, the MasterScreen PFT (Jaeger, CareFusion, Höchberg, Germany) was utilized. While in a standing position and wearing a nose clip, each participant took 5–6 resting tidal breaths before performing 10 s of maximal hyperventilation, with both large tidal volumes and a high respiratory frequency. Thereafter, the test was repeated 3–4 times, with a minimum two-minute break between each repetition. The highest value from two valid attempts with a difference of ≤3% was extrapolated to 60 s by multiplying the measured volume by six. This MVV value was used in the analyses. Some previous studies estimate MVV by multiplying FEV_1_ by 40, due to the excellent correlation between the two [31]. This was also performed in our study, but the former way of measuring MVV was the main approach used in the analyses.

VO_2_max was measured using a treadmill (ELG 90/200 Sports, Woodway, Weil am Rhein, Germany). Participant breathed through a three-way valve connected to a gas analyzer system (OxyconPro, Jaeger-Toennis, Höchberg, Germany) using the breath-by-breath method. Each participant began with a 15-min warm-up before the testing was initiated. The incline on the treadmill was set to 5%, and the pace was raised by 1 km/h every minute. The criteria for achieving VO_2_max were a combination of total exhaustion, a respiratory exchange ratio (RER) ≥ 1.1, and a plateau in the VO_2_ curve despite increasing the speed. The highest mean value of two consecutive 30-s measurements of VO_2_ determined the VO_2_max, which was expressed both in absolute values (L/min) and relative to body mass (mL/kg/min). During VO_2_max testing, V_E_max was also measured and defined as the mean ventilation per minute (L/min) at the end of exercise [3], understood as the point of total exhaustion.

Breathing reserve (BR) was calculated using the measured MVV and V_E_max for each participant. BR is expressed in both absolute values, defined as the difference between MVV and V_E_max (Equation (1)), and as a percentage derived from the ratio between V_E_max and MVV (Equation (2)). The latter is the most frequently used way of presenting BR in previous studies and is therefore also preferred in our study.

(1) $$BR\left(L/min\right)=MVV-V_{E}max$$

and

(2) $$BR\left(\%\right)=\left(1-\frac{V_{E}max}{MVV}\right)\times 100.$$

Statistical analyses in the present study were carried out using the software SigmaPlot 15 (Systat Software Inc., San Jose, CA, USA). Some of the data did not pass the Shapiro–Wilk normality test. Due to this, and the small sample size, significant differences between the runners and controls were tested using the Mann–Whitney rank-sum test. Effect size analyses were conducted with an online service for statistical analysis called AI-Therapy Statistics [32]. Additionally, a simple linear regression analysis was conducted to estimate the relationship between BR and VO_2_max, expressed as an unstandardized regression coefficient with confidence interval (CI). With this exception, all other data are presented as medians with 25% and 75% percentiles. The level of significance was set to p<0.05.

## 3. Results

### 3.1. Test Participants

As seen in Table 1, all anthropometrics, except for height, differed significantly between the two groups, all with *p* < 0.001. For instance, the median BMI was greater in the controls than in the elite runners, with [median (interquartile range)] [22.0 kg/m^2^ (20.7–24.5) vs. 19.7 kg/m^2^ (19.1–21.2)], respectively. As expected, the weekly training volume, both total and limited to only endurance training, was significantly higher in the group of elite runners, with a median difference in endurance training of 9.0 h per week (*p* < 0.001).

### 3.2. Lung Function

As shown in Table 2, there were no significant differences in the lung function parameters FEV_1_, FVC, and FEV_1_/FVC. All calculated effect sizes were below 0.3, indicating a small effect of endurance fitness level on lung function. All the mentioned parameters were also compared to the reference values according to the GLI-2012, indicating no sign of impaired lung function in any of the groups, nor any differences between them. All participants had values above LLN for all parameters, except for one person from each group for FEV_1_/FVC.

### 3.3. Breathing Reserve

**Figure 1:**
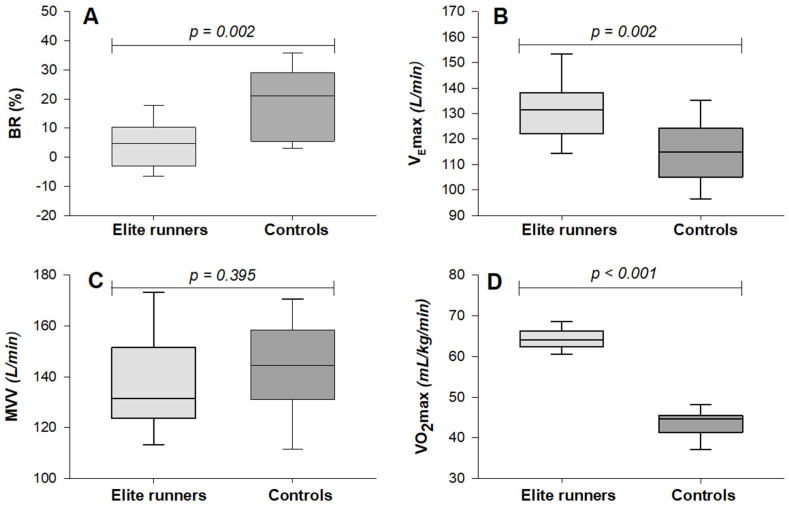
(**A**) BR as a percentage for elite runners and controls; (**B**) V_E_max; (**C**) MVV; (**D**) VO_2_max relative to body mass. Values are expressed as median with both 25–75% percentile and 10–90% percentile. Effect size, *r*, is 0.56 for BR, 0.56 for V_E_max, 0.16 for MVV, and 0.85 for VO_2_max. BR = breathing reserve; V_E_max = maximal exercise ventilation; MVV = maximal voluntary ventilation; VO_2_max = maximal oxygen uptake.

**Figure 2:**
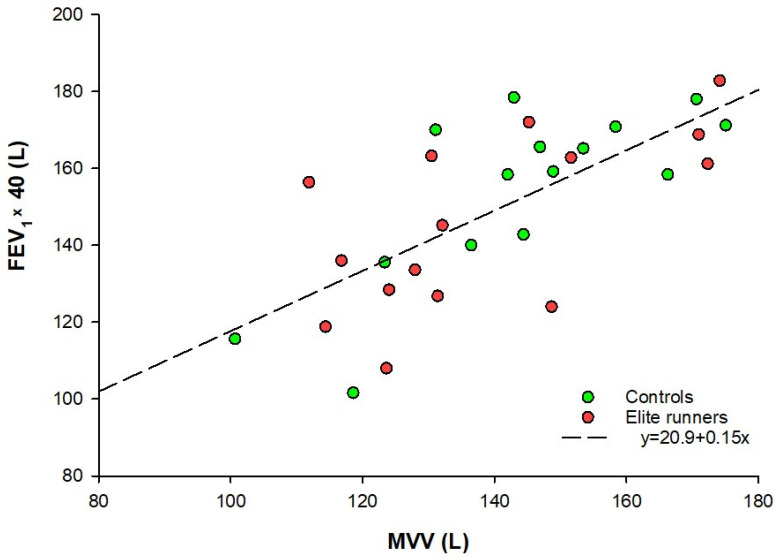
Scatterplot showing the relationship between measured MVV and MVV estimated by multiplying FEV_1_ by 40. MVV = maximal voluntary ventilation; FEV_1_ × 40 = forced expiratory volume during the first second multiplied by 40.

As seen in Figure 1, VO_2_max was significantly higher among the runners when measured relatively [64.0 mL/kg/min (62.4–66.2) vs. 44.7 mL/kg/min (41.4–45.5)], (p < 0.001). A greater VO_2_max in the group of runners was also seen when measured absolutely [3.7 L/min (3.4–3.9) vs. 2.9 L/min (2.7–3.0)], (p < 0.001). MVV did not show any significant difference between the groups, p = 0.395. However, V_E_max was significantly higher in the elite runners compared to the control group, with [131.5 L/min (122.1–138.3) vs. 114.8 L/min (105.1–124.3)], (p = 0.002), respectively. BR was significantly lower among the runners [4.8% (−3.1–10.3) vs. 21.2% (5.5–29.1)], (p = 0.002), with a median difference of 16.4 percentage points (8.6–18.8), as visualized in Figure 1. A high effect size of *r* = 0.56 supports a strong group difference in BR. Seven of the runners, and none of the controls, had a BR below 0%. BR calculated using MVV estimated from FEV_1_ showed the same pattern, with a smaller BR in elite runners [7.2% (0.8–19.6) vs. 26.2% (19.8–30.5)], (p < 0.001). The relationship between the MVV estimated this way and the measured MVV is illustrated in the scatterplot presented in Figure 2.

### 3.4. Breathing Reserve Dependent on Maximal Oxygen Uptake

**Figure 3:**
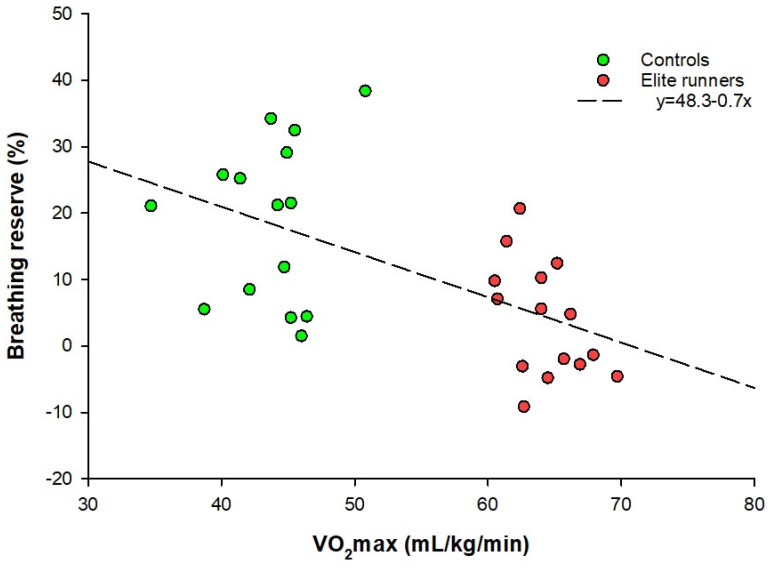
Scatterplot showing the relationship between BR as a percentage and VO_2_max relative to body mass. BR = breathing reserve; VO_2_max = maximal oxygen uptake.

The linear regression analysis results presented in Table 3 show that variation in the dependent variable BR can be explained by VO_2_max, with the latter being the independent variable, demonstrating p < 0.05 for all values. This applies to all combinations of BR measured as a percentage or L/min, and VO_2_max measured as an absolute or a relative value. However, the association is greater with VO_2_max measured relative to body mass compared to absolute VO_2_max values ($R^{2}$ = 0.34 and $R^{2}$ = 0.28 vs. $R^{2}$ = 0.28 and $R^{2}$ = 0.21). $R^{2}$, the explained variance, describes the proportion of variation in BR that can be explained by VO_2_max, expressed as a fraction. The highest $R^{2}$ value, 0.34, was obtained for the relative VO_2_max explaining BR as a percentage, where the unstandardized regression coefficient was found to be −0.7 percentage points (−1.1–−0.3). Figure 3 presents a scatterplot of BR expressed as a percentage versus relative VO_2_max, illustrating their observed association.

## 4. Discussion

Previous studies have reported breathing reserve in both trained and untrained populations, in addition to comparisons between genders. To our knowledge, none have compared a population of female elite runners to matched sedentary females. Thus, the findings of our study provide unique insight into the utilization of lung capacity during maximal exercise in these groups and may indirectly indicate whether the lungs can potentially limit elite runners’ performance. The main finding of our study is a significantly greater BR at maximal exercise in the group of young, healthy sedentary women compared to the runners. Hence, the runners utilize a greater proportion of their ventilatory capacity during intense exercise, suggesting that the pulmonary system is more likely to be a limiting factor for VO_2_max in this group. Unsurprisingly, the large unused proportion of ventilatory capacity among the untrained women generally confirms that the pulmonary system is not a limiting factor for their VO_2_max. Secondly, there is a moderate correlation between VO_2_max and BR, suggesting that factors other than VO_2_max influence BR. Finally, no significant differences in lung function, including FEV_1_ and FVC, were found between the two groups, indicating that lung function is not improved by endurance training.

The group of elite runners had a median of 10 weekly hours of endurance training, which was 9 h more than the controls. There was no difference in height, which is known to influence pulmonary function [19]. However, a great difference was seen in VO_2_max, with a median value of 64.0 mL/kg/min in the runners, approximately 20 mL/kg/min higher than in the controls.

A median BR of 21% in the control group, consisting of healthy, untrained women between the ages of 24 and 33, is within, but at the lower end of, the range reported in previous studies. These discrepancies may be partially explained by methodological differences, as well as variations in demographic or lifestyle characteristics of the control cohorts—such as age, cardiorespiratory fitness, ethnicity, or potential selection bias. Edvardsen et al. reported a mean BR of 30% for a subpopulation of women aged 20–29, predominantly Caucasian, with a VO_2_max of 40.3 mL/kg/min [4], while Habedank et al. reported a mean BR of 41% in a female population in the age range of 16–75 years [5]. Unlike our study, both studies investigated a mixed population of well-trained and untrained women. In addition, participants in the latter study had a mean VO_2_max of 32.2 mL/kg/min, which suggests that the study population was considerably worse in endurance training and probably had a higher median age than our study’s control group. This may explain why such a high BR was found. Blackie et al. investigated a more comparable subgroup to our controls, consisting of women aged 20–29 years, with competitive athletes being excluded [3]. They found a mean BR of 39%, which was also considerably higher than the finding in our study. However, VO_2_max was not reported for this subgroup, representing a potential source of bias.

Another methodological difference between our study and all three studies mentioned above is how MVV was assessed. Instead of measuring ventilation volume during maximal ventilatory effort for a given number of seconds and extrapolating to a minute, all three studies multiplied the measured FEV_1_ by 40 (Habedank et al. multiplied by 41 [5]). As mentioned earlier, this was performed because a previous study found an excellent correlation between FEV_1_ and MVV [31]. Kift and Williams measured MVV directly using the same method as our study, but they measured maximal ventilatory effort over 12 s, 30 s, and 60 s, and accordingly found three different MVVs [33]. Using the MVV extrapolated from the 12-s measurement, they reported a mean BR of only 11% for a population consisting of both males and females in their early 20s with a wide range of endurance fitness levels. McClaran et al. measured MVV in the same way in a group of women aged between 18 and 42 years, reporting a VO_2_max below 56 mL/kg/min (mean VO_2_max of 48.1 mL/kg/min), which was quite similar to the sedentary group in our study. They reported a mean BR of 21% [15], which was consistent with our findings. The implication of these different ways for assessing MVV will be discussed later. As discussed, the range of BR reported for untrained women is quite wide. However, the specific subgroups examined in our study have not been extensively investigated in previous studies, and therefore, our study provides valuable insights.

For the elite runners, a median BR of 5% was observed. No previous studies examining BR in female elite athletes were found, making the basis for comparison narrow. The only comparison that could be performed was with previous studies on male elite athletes. Generally, all previous studies examining BR in male athletes found a greater BR than that found in our study. Folinsbee et al. reported a mean BR of 11% for this group [6], and Lucia et al., in two different studies, reported means of 10–13% and 15%, respectively, in well-trained cyclists [11,12]. Åstrand & Rodahl found that very fit athletes could have a BR as low as 5% but provided no further details about what group of athletes was examined in this case [13]. This suggests that female elite athletes may have a smaller BR than matched men, which could be explained by their greater respiratory limitation due to smaller airways. However, it is important to emphasize that these are inferences based on BR and VEmax data, rather than direct evidence of physiological pulmonary limitation. Additionally, one should consider that the control group in our study exhibited a lower BR than what has been reported in most previous studies. This, combined with the low BR observed in the elite runners, may reflect methodological differences or potential selection bias.

A conclusion drawn from our findings is the significant difference in BR between the elite runners and the sedentary control group in our study. A difference of 16 percentage points was observed, and the large effect size of 0.56 indicates a strong group difference. This is in line with the findings of Folinsbee et al., who compared a group of well-trained males and untrained males, finding a BR of 11% and 29%, respectively [6]. To our knowledge, no previous studies have compared groups consisting only of women.

Few, if any, studies have explicitly examined the relationship between the degree of endurance fitness and BR. Blackie at al. concluded that V_E_max was closely related to VO_2_max [3], and because most studies, including this one, show that MVV is independent of fitness level, it could indicate that a correlation exists between BR and VO_2_max. Additionally, the fact that previous studies found a difference in BR between well-trained and untrained subgroups suggests that VO_2_max may be a predictive factor for BR. The present study found a correlation between the two factors, independent of whether VO_2_max was measured relative to body weight or in absolute values, and whether BR was measured as a percentage or as an absolute value. The strongest correlation was observed between relative VO_2_max and BR expressed as a percentage, wherein BR changes with −0.7 percentage points per 1 mL/kg/min increase in VO_2_max. Figure 3 visualizes this correlation. VO_2_max explains 34% of the variation in BR, demonstrating a moderate association; however, this result also indicates that other factors play a major role in explaining the variation in BR, as evidenced by the diverse values shown in Figure 3. Individual factors that influence the pulmonary physiology in general, such as height, BMI, genetics, and ethnicity, could be part of the explanation.

The controls had a higher median FEV_1_ as a percentage of the predicted value, and the elite runners had a higher median FVC as a percentage of the predicted value, but none of these differences were significant, and all effect sizes were low. This suggests that there was no difference in lung function between the two groups and supports the idea that the pulmonary system undergoes no major adaptations in response to endurance training. This is in line with the established understanding, as expressed by McArdle et al. [1]. Previous studies have reported conflicting findings in this field. Segizbaeva and Aleksandrova found significantly improved FEV_1_ and FVC in endurance athletes compared to healthy, less trained controls [24]. Nystad et al. found that decreased FEV_1_ was associated with decreased physical activity, after adjusting for factors such as smoking, sex, and height [22]. Furthermore, Twisk et al. found a significantly higher FVC in a physically active sample compared to an untrained sample [23]. Although we observed the same pattern in our study as that observed by Twisk et al., the lack of statistical significance strengthens the hypothesis that the difference in lung function is not determined by the degree of endurance capacity.

The present study has several limitations that should be considered when interpreting the results. Firstly, the examined sample, consisting of 15 elite runners and 15 controls, was relatively small, which may limit the generalizability of our findings and reduce the statistical power to detect minor differences or nuanced relationships. In addition, the controls were recruited from the same university, which makes it hard to strictly rule out any selection bias. Further, the inclusion of complementary measures such as arterial oxygen saturation, ventilatory thresholds, and respiratory muscle strength would have enhanced the physiological interpretation of the observed BR; however, these data were unfortunately not collected in the present study. Without these additional data, it is impossible to definitively determine whether a reduced BR significantly limits VO_2_max.

Finally, the measurement of MVV is, as previously mentioned, a much-discussed topic for several reasons. Measuring the maximal ventilation for a given time—in our study, 10 s—and then extrapolating to 1 min may give non-representative values. For instance, Kift and Williams compared the differences in estimated MVV based on measurements of 12, 30, and 60 s, finding a significant difference in estimated MVV between the measurements of 12 and 60 s [33]. When performing a 12-s measurement, they calculated a mean MVV of 115 L/min. In the 30-s and 60-s measurements, they estimated MVV values of 107 L/min and 102 L/min, respectively. These differences suggest that very short measurement durations can overestimate MVV since participants may be unable to sustain such high ventilatory intensity for an entire minute. In the present study, MVV was assessed over 10 s, which may have resulted in an overestimation of MVV and, thus, an overestimated breathing reserve (BR). Furthermore, Kift and Williams’ results showed a different breathing pattern when measuring the MVV compared to measuring ventilation during high-intensity exercise, with significantly lower breathing frequency and higher tidal volume obtained for the latter. This suggests that this way of measuring MVV may not estimate maximal ventilation in a reliable way, at least not when applied in training contexts. Short-term pulmonary adaptations, such as bronchodilation or bronchoconstriction, may occur during heavy exercise, but they may not occur to the same degree during MVV measurements, which is another factor that should be taken into account. Additionally, the fact that some studies measured MVV in this way and others estimated it based on FEV_1_ makes it difficult to compare across studies. Colwell and Bhatia further investigated this issue in a pediatric population and found a significant difference in calculated MVV when comparing the method of recording 12 s of ventilation and then extrapolating with the method of estimating MVV by multiplying FEV_1_ by 40 [8]. Using the different MVV values obtained to calculate BR, they found a significantly lower BR when MVV was measured compared to when it was estimated (12% vs. 36%). Figure 2 shows that, in our sample, MVV calculated from the 10-s measurements and MVV estimated as FEV_1_ multiplied by 40 were correlated. In our study, the difference in BR obtained from the two different methods of measuring MVV was not substantial, with MVV estimated from FEV_1_ yielding a BR of 26% for the controls and 7% for the runners. However, this absence of substantial difference does not necessarily apply to other studies due to methodical variations. At the same time, it is important to consider that the measurement of V_E_max is more reliable and standardized, and the fact that a difference in V_E_max observed in our study highlights that there probably is a real difference in BR between the groups. The runners in the present study had a significantly higher V_E_max of 15% (*p* = 0.002) compared to the controls, with medians of 132 L/min vs. 115 L/min, respectively. Nevertheless, greater emphasis should be placed on identifying a standardized way for measuring MVV in future research on this subject.

It would be interesting for future studies to examine the differences in BR between female athletes and healthy, untrained controls, as in our study, but with a larger sample showing greater heterogeneity. Comparing BR between male and female athletes, who are primarily from the same sports and fitness level percentiles, could provide insight into female respiratory limitations. In addition, measuring participants’ blood saturation for oxygen during maximal exercise testing may provide knowledge regarding whether a small BR may be a limiting factor for VO_2_max.

## 5. Conclusions

The elite runners participating in the present study were found to have a significantly smaller breathing reserve than young, healthy untrained women, and a correlation between VO_2_max and BR was evident. However, other factors also play a major role in determining the BR. The lung function parameters FEV_1_ and FVC did not differ between the two groups. The group of female elite runners demonstrated a smaller BR than that reported in previous studies of comparable male athletes, which may indicate a greater ventilatory demand in female endurance athletes compared to male endurance athletes. Larger study samples and the establishment of a standardized protocol for measuring MVV would be beneficial to further our knowledge and clarity on this subject.

## Figures and Tables

**Table 1:** Age, anthropometric data, and training volume for the elite long-distance runners and the controls.

Groups	Elite Runners (n = 15)	Controls (n = 15)	p-Value	Effect Size (*r*)
Age (years)	27.0 (25.0–30.0)	26.0 (24.0–28.0)	0.379	0.16
Height (cm)	169.5 (164.0–178.0)	173.0 (168.0–178.0)	0.261	0.21
Weight (kg)	55.8 (54.0–61.4)	64.5 (62.0–72.5)	<0.001	0.60
$\mathrm{BMI}\;(kg/m^{2}$)	19.7 (19.1–21.2)	22.0 (20.7–24.5)	<0.001	0.60
Fat mass (%)	16.5 (15.7–18.8)	29.5 (24.5–33.0)	<0.001	0.85
Training (h/w)	12.0 (11.0–15.0)	2.0 (1.0–3.5)	<0.001	0.85
Endurance training (h/w)	10.0 (9.0–15.0)	1.0 (0.0–1.0)	<0.001	0.86

Values expressed as median (25–75% percentile). BMI = body mass index; h/w = hours per week.

**Table 2:** Lung function measurements for the elite runners and controls.

	Groups	Elite Runners (n = 15)	Controls (n = 15)	p-Value	Effect Size (*r*)
FEV_1_	(L)	3.63 (3.17–4.08)	3.98 (3.50–4.27)	0.272	0.20
	(% of pred)	102 (93–111)	106 (97–112)	0.604	0.11
	Z-score	0.17 (−0.62–0.93)	0.52 (−0.29–1.07)	0.604	0.10
	LLN (n (%))	0 (0)	0 (0)	–	-
FVC	(L)	4.47 (3.98–4.85)	4.73 (4.11–5.04)	0.350	0.16
	(% of pred)	105 (98–111)	102 (100–113)	0.983	0.01
	Z-score	0.44 (−0.17–0.88)	0.20 (0.00–1.01)	0.983	0.06
	LLN (n (%))	0 (0)	0 (0)	–	-
FEV_1_/FVC	(%)	83.3 (77.9–84.8)	84.7 (79.0–88.6)	0.373	0.17
	(% of pred)	98 (91–100)	98 (93–104)	0.468	0.25
	Z-score	−0.25 (−1.20–0.02)	−0.26 (−0.94–0.55)	0.455	0.14
	LLN (n (%))	1 (6.7)	1 (6.7)	–	-

Values are expressed as median (25–75% percentile), except LLN, which is expressed as the number of people below LLN and their proportion in the total population. FEV_1_ = forced expiratory volume during the first second; FVC = forced vital capacity; pred = predicted value according to GLI-2012; Z-score = standard score; LLN = lower limits of normal.

**Table 3:** Regression analysis showing the association between BR (dependent variable) and VO_2_max (independent variable). All participants (n=30) were included in the regression analysis.

	VO_2_max (mL/kg/min)	$R^{2}$	p-Value	VO_2_max (L/min)	$R^{2}$	p-Value
BR (%)	−0.7 (−1.1–−0.3)	0.34	<0.001	−12.9 (−21.0–−4.8)	0.28	0.003
BR (L/min)	−1.0 (−1.6–−0.4)	0.28	0.003	−17.9 (−31.4–−4.5)	0.21	0.011

The results are presented as the unstandardized regression coefficient (b) with 95% CI and explained variance ($R^{2}$). BR = breathing reserve; VO_2_max = maximal oxygen uptake.

## Data Availability

The data presented in this study are openly available in the Supplemental Information as Supplementary Table S1. Raw data for age, height, and weight are not included to ensure the anonymity of the athletes.

## Supplementary Materials

The following supporting information can be downloaded at https://www.mdpi.com/article/10.3390/sports13070231/s1, Supplementary Table S1: Raw data.

## Abbreviations

The following abbreviations are used in this manuscript:

BR	Breathing reserve
FEV_1_	Forced expiratory volume in the first second
FVC	Forced vital capacity
VO_2_max	Maximal oxygen uptake
MVV	Maximal voluntary ventilation
V_E_max	Maximal minute ventilation
LLN	Lower limits of normal
BMI	Body mass index
